# Two Orders of Magnitude Variation of Diffusion-Enhanced Förster Resonance Energy Transfer in Polypeptide Chains

**DOI:** 10.3390/polym10101079

**Published:** 2018-09-29

**Authors:** Maik H. Jacob, Indrajit Ghosh, Roy N. D’Souza, Werner M. Nau

**Affiliations:** Department of Life Sciences and Chemistry, Jacobs University Bremen, 28759 Bremen, Germany; indrajit1.ghosh@ur.de (I.G.); r.dsouza@jacobs-university.de (R.N.D.)

**Keywords:** short-distance FRET, diffusion, radiative fluorescence lifetime, viscosity, chain dynamics, peptide structure

## Abstract

A flexible peptide chain displays structural and dynamic properties that correspond to its folding and biological activity. These properties are mirrored in intrachain site-to-site distances and diffusion coefficients of mutual site-to-site motion. Both distance distribution and diffusion determine the extent of Förster resonance energy transfer (FRET) between two sites labeled with a FRET donor and acceptor. The relatively large Förster radii of traditional FRET methods (*R*_0_ > 20 Å) lead to a fairly low contribution of diffusion. We introduced short-distance FRET (sdFRET) where Dbo, an asparagine residue conjugated to 2,3-diazabicyclo[2.2.2]octane, acts as acceptor paired with donors, such as naphtylalanine (NAla), tryptophan, 5-l-fluorotryptophan, or tyrosine. The Förster radii are always close to 10 Å, which makes sdFRET highly sensitive to diffusional motion. We recently found indications that the FRET enhancement caused by diffusion depends symmetrically on the product of the radiative fluorescence lifetime of the donor and the diffusion coefficient. In this study, we varied this product by two orders of magnitude, using both donors of different lifetime, NAla and FTrp, as well as a varying viscogen concentration, to corroborate this statement. We demonstrate the consequences of this relationship in evaluating the impact of viscogenic coadditives on peptide dimensions.

## 1. Introduction

Förster resonance energy transfer (FRET) brings the sensitivity and speed of fluorescence spectroscopy to structure and distance determination [1]. Since its first application to biopolymers, namely polyprolines [2], major efforts have been invested to not only broaden the range of FRET applications, but to also consolidate and enhance the information it provides [3,4,5,6]. Although single-molecule FRET is certainly one of the most notable outcomes of these efforts [3,7,8,9], developments in ensemble-FRET technology also deserve attention. This article is about one of them.

The major parameter that characterizes any FRET method is the Förster radius, the distance where the probability that the donor becomes deactivated by photon emission and external quenching is as likely as its deactivation by energy transfer to the acceptor [1]. As donor-acceptor distances become shorter, the probability of FRET events increases with the sixth power. Large Förster radii are suitable to detect large distances; small radii are required if short distances and minute distance differences have to be detected. We introduced short-distance FRET (sdFRET), where Dbo, an asparagine residue conjugated to 2,3-diazabicyclo[2.2.2]octane, acts as an acceptor paired with donors, such as naphtylalanine (NAla), tryptophan, 5-fluorotryptophan, or tyrosine [10,11,12,13]. The Förster radii are always about 10 Å, which is about half of the lower limit offered by traditional donor-acceptor pairs. The high sensitivity towards small distance differences brings about a particularity of sdFRET not seen with any other FRET method [13].

Each donor is characterized by its natural fluorescence lifetime, the reciprocal decay rate in the absence of an acceptor. Depending on this lifetime, the donor can shorten its distance to the acceptor by Brownian motion, and, thereby, increase the probability of FRET events [6,14]. This mutual diffusional motion between labeled chain positions enhances FRET by a quantity known as FRET diffusion enhancement (FDE) [6,13]. The parameters of the molecular system that determine the extent of FRET are the distances between donors and acceptors, as captured by the equilibrium distance distribution, as well as their end-to-end motion, usually captured by a single diffusion coefficient. In traditional FRET methods, the impact of diffusion is often treated as negligible; the question whether this treatment is permissible should become an open debate. In sdFRET, the FDE is simply too obvious to be neglected, and it must be considered to obtain meaningful information. The parameters of the photophysical probes that determine the extent of FRET are the Förster radius and the donor lifetime. However, we have recently shown that FDE cannot be reduced, as had been proposed [15], by simply adding a co-agent to the experimental solutions that quenches the donor and reduces its lifetime [13]. Based on theoretical arguments, experiments, and simulations, we concluded that it is not the experimentally measured lifetime, but the radiative donor lifetime (the lifetime extrapolated to a donor quantum yield of unity), which determines the FDE. Thus, the view that short donor lifetimes allow diffusion to be neglected should indeed be revisited for every particular system.

**Figure 1:**
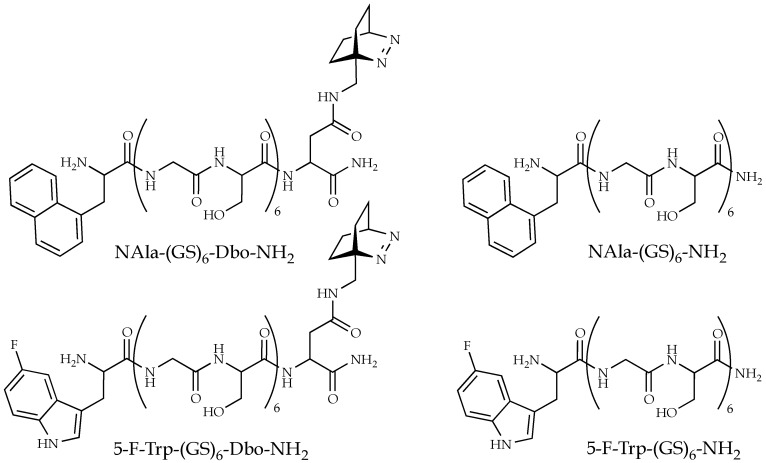
The selected donor-acceptor and donor-only labeled peptides composed of Gly-Ser units. Donors are N-terminal naphthyl-1-l-alanine (NAla) and 5-fluoro-l-tryptophan (5-F-Trp); the acceptor is C-terminal Dbo, whose optically active group is the azo group of the bicyclic chromophore.

Preliminary experiments in the absence and presence of ethylene glycol, a viscogenic agent, led us to the hypothesis that what determines the FDE is the product of the radiative lifetime and the end-to-end diffusion coefficient. Here, we put this assumption of such a symmetry between the radiative lifetime and diffusion on solid ground. We were indeed able to vary this product over a large range by two orders of magnitude, and used, to that end, labeled Gly-Ser repeat peptides, (GS)_6_, with two donors of largely different radiative lifetimes, NAla and FTrp. The peptides were sufficiently long to avoid steric-hindrance effects and sufficiently short to lead to sizable FRET (Figure 1). In addition, we varied the end-to-end diffusion coefficient by altering the ethylene glycol content of the solutions. As a viscogen, ethylene glycol allows practical experiments to be done at very high concentrations and guarantees, because of its small size, that the measured macroviscosity is closest to microviscosity relevant on a molecular scale. Because of these properties, ethylene glycol has previously proven instrumental in elementary questions on protein folding [16,17,18,19]. The theoretical framework of the analysis is found in the Materials and Methods because the foundations have been laid in previous studies [1,13,20].

## 2. Materials and Methods

Donor-acceptor and donor-only labeled peptides (Figure 1) were obtained in >95% purity (Biosyntan, Berlin, Germany). All peptides were amidated at the C terminus to exclude attractive electrostatic interactions between the terminal positions. Fmoc-Dbo and 5-fluoro-l-tryptophan required for peptide synthesis were prepared according to literature methods [21,22]. Other chemicals were from Sigma (Munich, Germany). Absorption spectra were recorded on a Cary 4000 UV–Vis spectrophotometer (Varian, Santa Clara, CA, USA) and steady-state fluorescence spectra on a Cary Eclipse fluorimeter (Varian). Time-resolved fluorescence decays were recorded on a time-correlated single-photon-counting instrument (FLS920, Edinburgh Instruments, Livingston, UK) by using a pulsed diode laser (Picoquant, Berlin, Germany) for excitation at 280 nm in the NAla and FTrp sdFRET measurements. Peptide concentrations were determined by using extinction coefficients of 5.7 × 10^3^ M^−1^ cm^−1^ (FTrp) and 5.5 × 10^3^ M^−1^ cm^−1^ (NAla) at 280 nm; they were adjusted to 10 µM in aerated solutions of varying ethylene glycol content, 25 °C, pH 5.0. The donor quantum yields were determined by comparison with *N*-acetyl-tryptophanamid (0.14, pH 7.0) [23,24,25]. The Förster radii of NAla/Dbo and FTrp/Dbo were determined by using the absorption and emission spectra of the single-labeled peptides as previously described [11,23].

Fluorescence lifetime decay traces in the presence of FRET were obtained from the donor-acceptor labeled peptides, NAla-(Gly-Ser)_6_-Dbo-NH_2_ and 5-F-Trp-(Gly-Ser)_6_-Dbo-NH_2_, and, in the absence of FRET, from the donor-only peptides, NAla-(Gly-Ser)_6_-NH_2_ and 5-F-Trp-(Gly-Ser)_6_-NH_2_. Upon excitation of NAla or FTrp at 280 nm and emission recording at 350 nm, the traces were analyzed by using the FLS920 instrument software (Edinburgh Instruments, Livingston, UK) as described [23] and by using the software ProFit (Quantumsoft, Zürich, Switzerland). The reproducibility of the reported fluorescence lifetimes for the monoexponential decays was ±3%.

## 3. Theoretical Analysis

This study was designed to test whether the FDE depends symmetrically on the radiative donor lifetime and the end-to-end diffusion coefficient. The effective distance treated in the following depends only on the equilibrium distance distribution and the FDE; it decreases with increasing FDE and increases with decreasing FDE. As the end-to-end distance distributions of NAla- and FTrp-labeled peptides can be assumed to be identical, the effective distance changes only in dependence on FDE. Therefore, we can test whether FDE really depends only on the product of the diffusion coefficient and the radiative lifetime (*Dτ*_rad_) by plotting it as a function of *Dτ*_rad_, or as a function of the product of the inverse viscosity times the inverse radiative lifetime or intrinsic decay rate (*Dτ*_rad_ ∝ *η*^−1^*k*_rad_). Towards this goal, it will not be sufficient to only change the ethylene glycol content. To be convincing, we had to switch donors to achieve dramatically different radiative lifetimes that, together with the variation in viscosity, would allow us to cover two orders of magnitude changes in *Dτ*_rad_. In the following, we outline the analysis that yielded the effective distances and the products of the inverse viscosities and radiative lifetimes.

(a) The effective distance

The measured fluorescence lifetimes of the donor-acceptor-labeled peptides, *τ*_DA_, and of the donor-only peptides, *τ*_D_, were converted into the corresponding decay rates, *k*_D_ and *k*_DA_, according to Equation (1), which yielded the FRET rate constant, *k*_FRET_, according to Equation (2).

(1) $$k_{D}=\tau_{D}{}^{-1}\text{ and }k_{\mathrm{DA}}=\tau_{\mathrm{DA}}{}^{-1}$$

(2) $$k_{\mathrm{FRET}}=k_{\mathrm{DA}}-k_{D}$$

At all ethylene glycol concentrations, the kinetic traces followed monoexponential time courses. In the case of the FTrp-labeled peptide at high ethylene glycol concentrations, biphasic fits seemed to be slightly more appropriate, and were used to calculate amplitude-weighted lifetimes, $\tau =\sum_{i}A_{i}\tau_{i}$, in accordance with Ref. [4]. The effective distance is defined by Equation (3), which has the form of Förster’s law (Equation (4)). The effective distances were calculated from Equation (5).

(3) $$k_{\mathrm{FRET}}=k_{D}{\left(\frac{R_{0}}{R_{\mathrm{eff}}}\right)}^{6}$$

(4) $$k_{T}(r)=k_{D}{\left(\frac{R_{0}}{r}\right)}^{6}$$

(5) $$R_{\mathrm{eff}}=k_{D}{\left(\frac{k_{D}}{k_{\mathrm{FRET}}}\right)}^{1/6}R_{0}$$

The Förster radius increases slightly with the ethylene glycol concentration because ethylene glycol increases the refractive index of the experimental solutions as well as the donor quantum yield of the experimental peptides. The experimental quantum yields were obtained as follows: The decay rate of the donor is the sum of the radiative and the non-radiative decay rate in the donor-only peptide (Equation (6)).

(6) $$k_{D}=k_{\mathrm{rad}}+k_{\mathrm{nrad}}$$

The donor quantum yield, Φ_D_, is the ratio of the radiative decay rate, *k*_rad_, and the decay rate, *k*_D_ (Equation (7)). The values of the quantum yield and radiative decay rate in the absence of ethylene glycol have been reported [13].

(7) $$\Phi_{D}=\frac{k_{\mathrm{rad}}}{k_{\mathrm{rad}}+k_{\mathrm{nrad}}}=\frac{k_{\mathrm{rad}}}{k_{D}}$$

The radiative decay rate depends on the refractive index, *n*, of the experimental solution, which increases slightly with the ethylene glycol concentration (Equation (8)) [20,26]. The refractive index in the absence of ethylene glycol, *n*_0_, equals 1.3328 under our conditions.

(8) $$k_{\mathrm{rad}}\left(n\right)=k_{\mathrm{rad}}\left(n_{0}\right)\cdot \frac{n^{2}}{n_{0}^{2}}$$

Accordingly, the quantum yields were calculated from Equation (9):

(9) $$\Phi_{D}\left(n\right)=\frac{k_{\mathrm{rad}}(n)}{k_{D}(n)}=\frac{k_{\mathrm{rad}}\left(n_{0}\right)}{k_{D}(n)}\frac{n^{2}}{n_{0}^{2}}$$

How the Förster radii depend on the quantum yields and the refractive indices is captured in Equations (10) and (11). The Förster radii of the FTrp/Dbo and the NAla/Dbo donor/acceptor pair in the (GS)_6_ peptides in water, $R_{0}\left(\Phi_{D,n_{0}},n_{0}\right)$, have been reported (FTrp/Dbo: *R*_0_ = 9.6 Å; NAla/Dbo: *R*_0_ = 9.8 Å).

(10) $$R_{0}^{6}\left(\Phi_{D},n\right)=C\left(\Phi_{D},n\right)\cdot R_{0}^{6}\left(\Phi_{D,n_{0}},n_{0}\right)$$

The correction term, *C*, is calculated from Equation (11):

(11) $$C\left(\Phi_{D},n\right)\equiv \frac{\Phi_{D}}{\Phi_{D,n_{0}}}\frac{n_{0}^{4}}{n^{4}}$$

Accordingly, the Förster radii at a given ethylene glycol concentration were calculated from Equation (12):

(12) $$R_{0}\left(\Phi_{D},n\right)=C{\left(\Phi_{D},n\right)}^{1/6}\cdot R_{0}\left(\Phi_{D,n_{0}},n_{0}\right)$$

When the resulting $R_{0}\left(\Phi_{D},n\right)$ values are entered in Equation (5), the effective distances are obtained.

The Förster radius, *R*_0_, is also proportional to the sixth root of the orientation factor, *κ*^2^. The factor, *κ*^2^, captures that the dipole moments of the donor and acceptor can adopt various orientations towards each other and can vary between 0 (perpendicular vectors) and 4 (collinear vectors). The consequences of possibly misjudging the value of *κ*^2^ were recently discussed [27]. The orientation factor adopts a value of 0.67 when donor and acceptor dipoles sample all possible orientations randomly and rapidly in comparison to the time scale of the donor emission decay [28]; this is the usual assumption that we also employed. However, it adopts the slightly smaller value of 0.48, when dipole orientations are random, but remain virtually frozen during the radiative donor lifetime [29,30], which translates (due to the sixth root dependence) into 5% smaller Förster radii and effective distances. However, we recently applied sdFRET to short polyproline peptides and could show by MD simulations that probe orientations are randomized due to the flexible linkers that connect the optically active probes to the polyproline chain [10]. Furthermore, the probes we employ in sdFRET are small and the time of randomization, the reorientation time, is well within the picosecond time scale [31]. Thus, even if, in the presence of a viscogen, the reorientation time is increased by an order of magnitude, it is still small compared to the donor lifetime (NAla, 256 ns; FTrp, 19.8 ns), and the criteria discussed in reference [27] are met.

(b) The product of radiative lifetime and diffusion coefficient

The assumption to be tested was that the FDE depends symmetrically on the radiative lifetime, *τ*_rad_, (Equation (13)), and the diffusion coefficient, *D*; that is, on the product, *Dτ*_rad_. We assumed that the diffusion coefficient is proportional to the reciprocal viscosity (Equation (14)). The radiative lifetimes have been determined as outlined above. If the effective distance depends symmetrically on the diffusion coefficient and the radiative lifetime (Equation (15)), it also depends symmetrically on the viscosity and radiative rate (Equation (16)), and vice versa. The relationship between the concentration of aqueous ethylene glycol solutions and their viscosity has been established in Ref. [32] (Equation (17)), where *x*_EG_ is the mole fraction of ethylene glycol.

(13) $$\tau_{\mathrm{rad}}=k_{\mathrm{rad}}^{-1}$$

(14) $$D\propto \eta^{-1}$$

(15) $$R_{\mathrm{eff}}=f(D\tau_{\mathrm{rad}})$$

(16) $$R_{\mathrm{eff}}=f(\eta k_{\mathrm{rad}})$$

(17) $$\eta =0.8938+7.4579x_{EG}+13.3928x^{2}{}_{EG}-4.7608x^{3}{}_{EG}$$

(c) The end-to-end diffusion coefficient and distance probability distribution obtained from a global analysis based on the Haas-Steinberg equation

The Haas-Steinberg equation (HSE, Equation(18)), first derived in reference [14], relates the rate of donor deactivation—the decrease with time of the number, N*, of chains with an excited donor in dependence of the donor-acceptor distance, N*(*r,t*)/∂*t*, in donor-acceptor labeled chains to the sum of three terms accounting for the donor decay in the absence of FRET, for the donor decay caused by FRET, and for diffusion.

(18) $$\frac{\partial N^{*}\left(r,t\right)}{\partial t}=-k_{D}N^{*}\left(r,t\right)-k_{D}\frac{R_{0}^{6}}{r^{6}}N^{*}\left(r,t\right)+\frac{\partial }{\partial r}\left(N_{0}^{*}\left(r\right)D\frac{\partial \left(N^{*}/N_{0}^{*}\right)}{\partial r}\right)$$

The initial distance distribution, *N*_0_*(*r*) or *N*_0_*, instantly after short-pulse donor excitation, mirrors the distance distribution in all peptide chains in the experimental sample because the probability of donor excitation does not depend on the distance of the acceptor. Thus, *N*_0_*(*r*), when normalized to ∫*N*_0_*(*r*) d*r* = 1, is identical to the probability distribution in the ground-state equilibrium ensemble of chains, i.e., *N*_0_*(*r*) = *p*(*r*). In the widely used skewed Gaussian distribution (Equation (19)), the meaningful parameters, *a* and *b*, determine the shape of the distribution, whereas *c* is a normalization constant determined by the condition that the integral of the probability density over all possible distances has to equal unity, ∫*p*(*r*) d*r* = 1.

(19) $$p(r)=c\cdot \pi r^{2}\exp(-a{\left(r-b\right)}^{2})$$

In essence, when the photophysical parameters, the donor decay rate constant in absence of the acceptor, *k*_D_, the Förster radius, *R*_0_, as well as the parameters describing chain conformation and dynamics, *a*, *b*, and *D*, are fed into the HSE—a linear partial differential equation, which can only be solved numerically, the HSE returns the fluorescence decay kinetics and, through further calculation, previously outlined in reference [13], the effective distance, *R*_eff_. As we determined the *k*_D_ and *R*_0_ values for 48 donor/viscosity combinations, we could simultaneously analyze 48 equations, each with its own pair of *k*_D_ and *R*_0_ values, with the diffusion coefficient, *D_η_*, at viscosity, *η*, given by Equation (20)—where *D* and *η*_0_ are the values in water—and with the parameters of the distance distribution, *a* and *b*, whose values were kept constant in all 48 equations.

(20) $$D_{\eta }=D\cdot \frac{\eta_{0}}{\eta }$$

In the global optimization, *a*, *b*, and *D* were varied and the equations solved till the sum-of-squares difference between the computed and the experimental *R*_eff_ values could not be further minimized. The result is illustrated in Figure 7 in the Results and Discussion section. This kind of optimization does not directly provide standard deviations of the parameters of interest. Therefore, we further tested the reliability of the obtained values by performing the same global optimization analysis on subsets that contained only a limited number (15 to 35) of data points, randomly chosen from the set of 48 experimental data points (200 optimizations). The computations were carried out in MATLAB (MathWorks).

## 4. Results and Discussion

**Figure 2:**
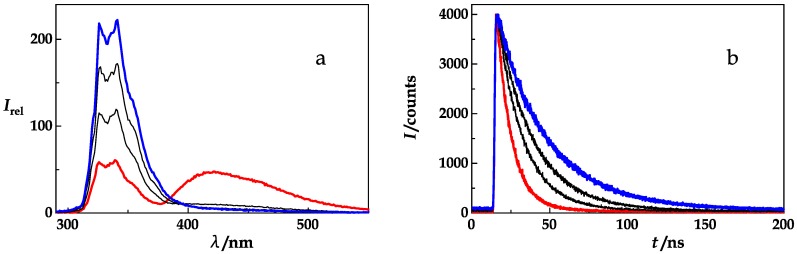
(**a**) Steady-state fluorescence emission spectra and (**b**) time-resolved fluorescence decays (*λ*_obs_ = 350 nm) of NAla-(GS)_6_-Dbo-NH_2_ upon excitation at 280 nm at ethylene glycol concentrations (vol/vol) ranging from 0% (red) to ca. 92% (blue). The donor lifetime increases from 10.3 ns (red curve) to 34.3 ns (blue curve).

That FRET in the double labeled (GS)_6_ peptides occurs and how it is strongly influenced by ethylene glycol is illustrated in Figure 2. The peptide, NAla-(GS)_6_-Dbo-NH_2_, shows a fluorescence spectrum (red curve) with two regions of maximal intensity, one around 340 nm arising from NAla fluorescence and another at 440 nm due to Dbo fluorescence. Since Dbo in the absence of a donor cannot be optically excited at 280 nm [12], it receives its excitation from NAla through FRET. In the presence of increasing concentrations of ethylene glycol, the fluorescence from NAla increases and the fluorescence from Dbo decreases. This is a first indication of reduced diffusion, and of a reduced FDE in the presence of viscogen. A second cause is the well documented quenching of Dbo by protic solvents [33].

In Figure 2b, time-resolved measurements show an increase of the donor (NAla) lifetime in the presence of FRET and in the presence of an increasing concentration of ethylene glycol, which parallels the steady-state fluorescence increase in Figure 2a. It was possible and beneficial to base the quantitative analysis (see Materials and Methods) on time-resolved measurements as they do not depend on the absolute peptide concentration.

**Figure 3:**
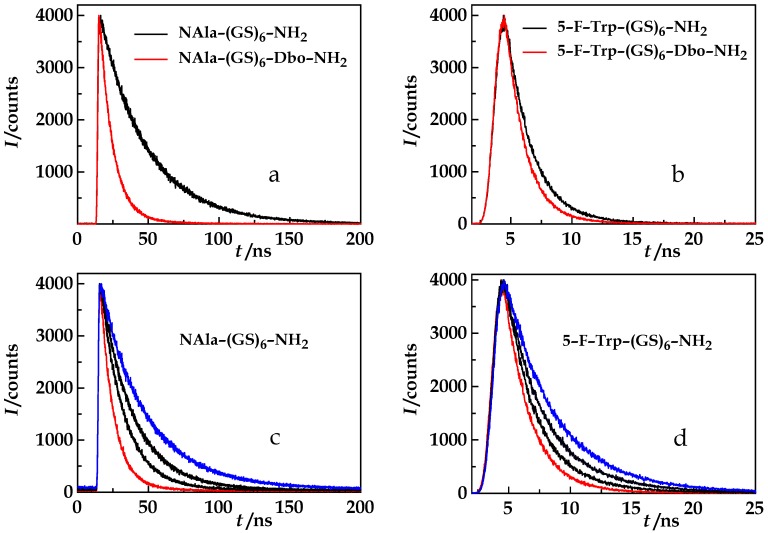
(**a**) NAla sdFRET measurements: Time-resolved fluorescence decays measured with NAla-(GS)_6_-NH_2_ (black) and NAla-(GS)_6_-Dbo-NH_2_ (red) at 350 nm after excitation at 280 nm in water. The lifetime of the donor-acceptor peptide, *τ*_DA_ = 10.3 ns, and the lifetime of the donor-only peptide, *τ*_D_ = 34.7 ns, yield an energy transfer efficiency of 70.4% and an effective distance of 8.5 Å. (**b**) FTrp sdFRET measurements: Time-resolved fluorescence decays measured with 5-F-Trp-(GS)_6_-NH_2_ (black) and 5-F-Trp-(GS)_6_-Dbo-NH_2_ (red) at 350 nm after excitation at 280 nm in water. The lifetime of the donor-acceptor peptide, 2.0 ns, and the lifetime of the donor-only peptide, 1.5 ns, yield an energy transfer efficiency of 24.7% and an effective distance of 11.5 Å. (**c**) Time-resolved fluorescence decays measured with NAla-(GS)_6_-Dbo-NH_2_ and (**d**) 5-F-Trp-(GS)_6_-Dbo-NH_2_ at 350 nm after excitation at 280 nm, at ethylene glycol concentrations (vol/vol) ranging from 0% (red) to 92% (blue).

Figure 3a,b show the measurements that form the basis of the NAla and FTrp sdFRET analysis. The obtained lifetimes of the donor-acceptor and donor-only labeled peptides yield the FRET quenching rates as expressed in Equations (1) and (2). The radiative lifetime, *τ*_rad_, of the NAla-only peptide (256 ns, *τ*_rad_ = *τ*_D_/Φ_D_) exceeds the radiative lifetime of the FTrp-only peptide (19.8 ns) by a factor of 13. While, in the presence of an acceptor, FRET shortens the donor lifetime (red traces in Figure 3a,b), ethylene glycol increases it by increasing the quantum yield of both donors, NAla and FTrp (Figure 3c,d).

**Figure 4:**
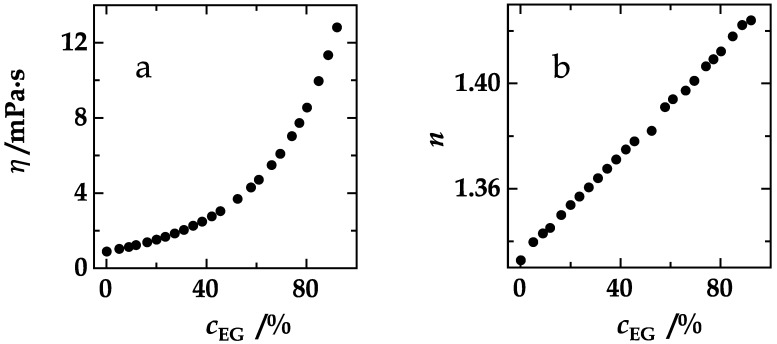

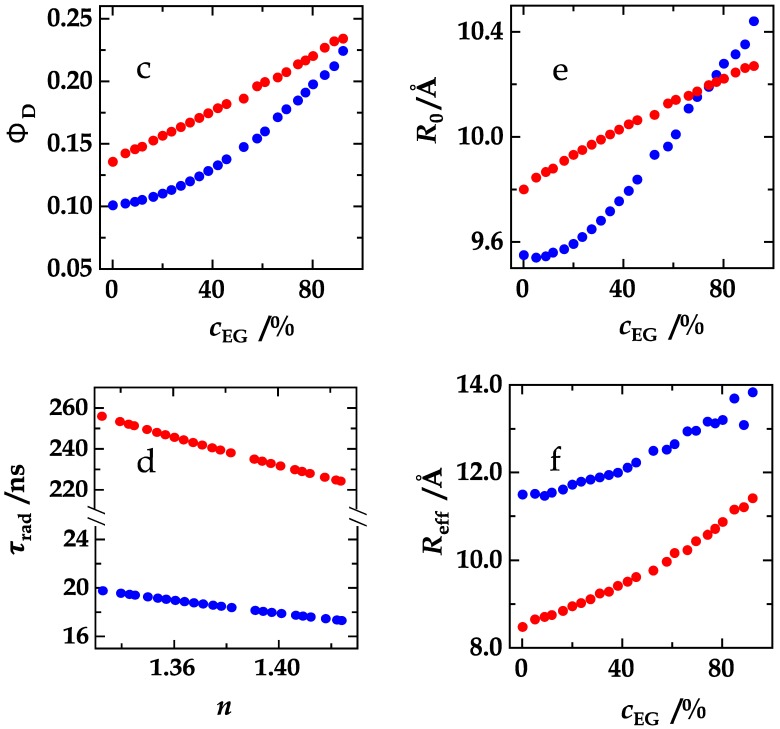
(**a**) Viscosity and (**b**) refractive index of aqueous ethylene glycol solutions plotted against the ethylene glycol concentration. (**c**) Donor quantum yield of NAla in NAla-(GS)_6_-NH_2_ (red) and of 5-F-Trp in 5-F-Trp-(GS)_6_-NH_2_ (blue) plotted against ethylene glycol concentration. (**d**) Radiative lifetimes of NAla in NAla-(GS)_6_-NH_2_ (red) and of 5-F-Trp in 5-F-Trp-(GS)_6_-NH_2_ (blue) plotted against the refractive indices that correspond to the ethylene glycol concentrations shown in (**b**). (**e**) Förster radii of the NAla/Dbo donor/acceptor pair in NAla-(GS)_6_-Dbo-NH_2_ (red) and of the 5-F-Trp/Dbo donor/acceptor pair in 5-F-Trp-(GS)_6_-Dbo-NH_2_ (blue) plotted against ethylene glycol concentration. (**f**) The effective donor/acceptor distances in NAla-(GS)_6_-Dbo-NH_2_ (red) and in 5-F-Trp-(GS)_6_-Dbo-NH_2_ (blue) plotted against the ethylene glycol concentration.

In a compilation, Figure 4 visualizes the experimental data required to reveal the relationship between the effective distance and the product that, as we hypothesized, solely determines the FDE, the product, *D*𝜏_rad_, or, eqivalently, *ηk*_rad_ (Equations (13)–(16)). The viscosity of the experimental solutions increases exponentially with the ethylene glycol concentration (Figure 4a, Equation (17)) and the refractive index linearly (Figure 4b). The refractive index, in turn, affects the radiative lifetimes of the donors, NAla and FTrp (Figure 4d), according to Equation (8). Donor quantum yields were calculated according to Equation (7). The quantum yield of NAla increases with ethylene glycol content (Figure 4c, red). The quantum yield of FTrp (Figure 4c, blue) increases even more and almost coincides with that of NAla at the highest ethylene glycol concentration that we employed (92%). This stronger dependence of the FTrp quantum yield on the presence of the coadditive is also reflected in the case of the Förster radii (Equations (10)–(12)) of the donor/acceptor pairs, NAla/Dbo (red) and FTrp/Dbo (blue), shown in Figure 4e. While both graphs show an increase of *R*_0_, they do not run in parallel. At high viscogen concentrations, the FTrp/Dbo values even exceed the NAla/Dbo Förster radii. The effective donor/acceptor distances in NAla-(GS)_6_-Dbo-NH_2_ (red) and in 5-F-Trp-(GS)_6_-Dbo-NH_2_ (blue) could now be obtained from Equation (5) (Figure 4f).

**Figure 5:**
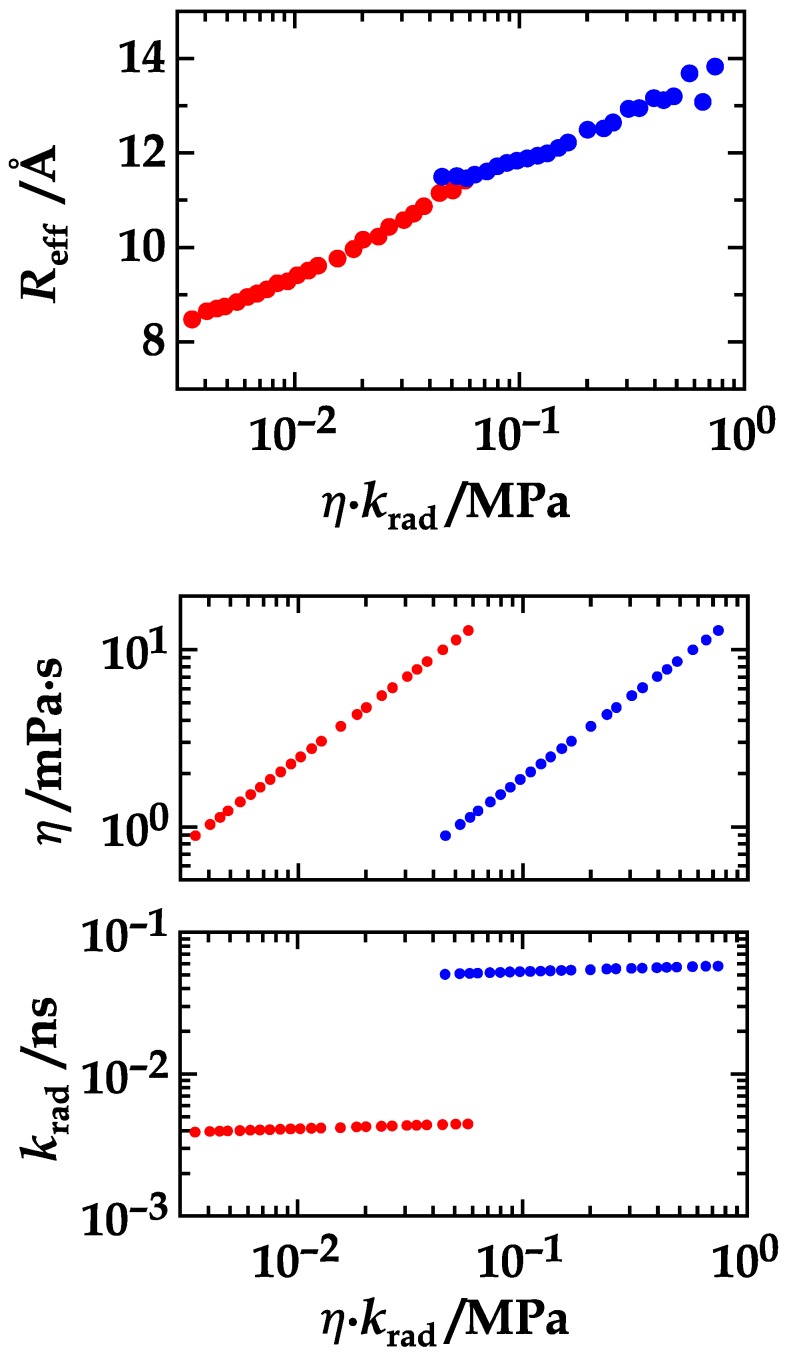
(**Top panel**) The effective donor/acceptor distance in NAla-(GS)_6_-Dbo-NH_2_ (red) and 5-F-Trp-(GS)_6_-Dbo-NH_2_ (blue) plotted against the product, *ηk*_rad_, of the viscosity of the aqueous ethylene glycol solutions and the intrinsic decay rates (reciprocal radiative lifetimes) of NAla in NAla-(GS)_6_-NH_2_ and of 5-F-Trp in 5-F-Trp-(GS)_6_-NH_2_ (blue). (**Middle panel**) The corresponding viscosity values and (**Bottom panel**) radiative decay rates of NAla in NAla-(GS)_6_-NH_2_ (red) and of 5-F-Trp in 5-F-Trp-(GS)_6_-NH_2_ (blue).

With the combined data in hand, it is possible to retrieve the relationship between the effective distance and the product, *ηk*_rad_, of viscosity, *η*, and inverse radiative lifetime, *k*_rad_ (Figure 5, top). The inverse product, (*ηk*_rad_)^−1^, is directly proportional to *D*𝜏_rad_ (Equations (13)–(16)). To ease the understanding of Figure 5, we depicted the courses of its components, the viscosity and the inverse lifetime, beneath it (Figure 5, middle and bottom panel). The critical step is going from solutions that contain high amounts of ethylene glycol and peptides labeled with NAla or NAla/Dbo to solutions that contain no or little amounts of ethylene glycol and peptides labeled with FTrp or FTrp/Dbo. This jump in viscosity when going from ethylene glycol to water is perfectly balanced when simultaneously going from NAla to FTrp. The continuity of the course of the effective distance proves that it is only the product, *ηk*_rad_, and, equivalently, *D*𝜏_rad_, which influences the FDE. With a decrasing FDE (Figure 5), the effective distance increases from ca. 8 Å to ca. 14 Å. Our choice of (GS)_6_ model peptides seemed to be appropriate as their dimensions emerged to be a good match to the Förster radii (ca. 10 Å) of the sdFRET methods. The remarkable increase of the effective distance emphasizes that any FRET-based study and inferrence on peptide structure could be gravely incorrect if a possible FDE is simply ignored.

**Figure 6:**
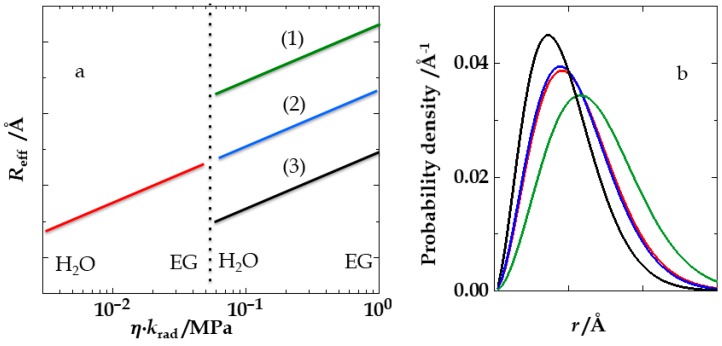
(**a**) The effective donor-acceptor distance (*R*_eff_, relative scale) in donor/acceptor labeled peptides plotted against *ηk*_rad_. The dotted vertical line marks the point where the viscosity switches back from high to low ethylene glycol content and where a donor of high radiative lifetime is exchanged for one with low radiative lifetime. The three possible cases are (1), (2), and (3). (1) The end-to-end distances in the experimental peptide shorten upon addition of ethylene glycol, such that the switch back to low ethylene glycol content shows an up-jump of the effective distance (red and green line). (2) The distances are completely independent of the ethylene glycol concentration (red and blue line). (3) The peptide expands upon addition of ethylene glycol, such that the switch back to low ethylene glycol content shows a down-jump of the effective distance (red and black line). (**b**) The distance distributions (probability density plotted against relative donor-acceptor distance) that correspond to the three cases in (**a**) shown in corresponding colors. For instance, case (2) points to virtually identical distance distributions (red and blue) that do not change with the ethylene glycol concentration.

The *Dτ*_rad_ or *ηk*_rad_ symmetry has important implications. In Figure 6, the effective distance is again plotted against *ηk*_rad_. The dotted line marks the simultaneous switch from high to low viscosity and from a short to a long donor lifetime. If the coadditive has no impact on peptide dimensions, this course is continuous (case (2)). If the intrachain distances contract upon addition of the coadditive, they should expand again when the experimental series goes from highest to lowest ethylene glycol content (case (1)). If the intrachain distances expand upon addition of the coadditive, they should contract again when the experimental series starts again at 0% ethylene glycol (case (3)). In this study, we clearly observed a type-2 course. Even though ethylene glycol changes not only the viscosity and the refractive index of the solutions, but also the dielectric permittivity and other properties, it has, even at very high concentrations, no impact on the peptide dimensions.

How a coadditive influences peptide dimensions is a topic of interest to the protein folding community and in the field of natively unfolded proteins or intrinsically disordered peptides [34,35]. The depicted procedure—the simultaneous switch of viscosity and donor lifetime—could be transferred to investigations of the impact of other viscous coadditives (urea, guanidinium chloride, or glycerin) on the dimensions of peptide chains, in which the number and distribution of ionizable, polar and hydrophobic residues could also be varied.

**Figure 7:**
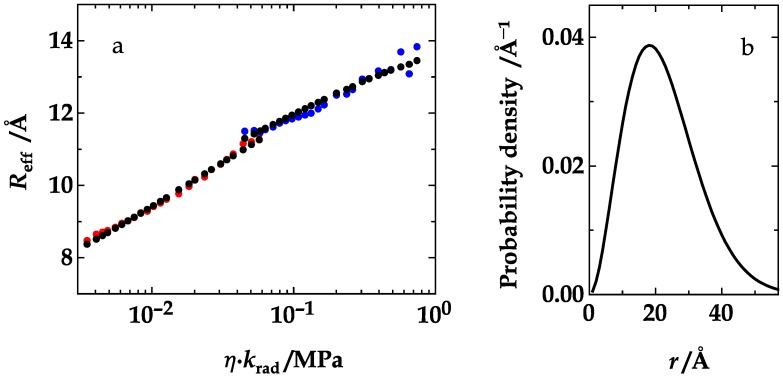
(**a**) The effective donor/acceptor distance in NAla-(GS)_6_-Dbo-NH_2_ (red) and 5-F-Trp-(GS)_6_-Dbo-NH_2_ (blue) plotted against the product, *ηk*_rad_, as in the top panel of Figure 5. The black circles represent the best fit of the data points, as obtained from an optimization based on the numerical solution of the Haas-Steinberg equation (HSE, Equation (18)). It yielded a diffusion coefficient of 55.4 Å^2^/ns in the absence of ethylene glycol, and a skewed Gaussian distance distribution, *p*(*r*) = *cr*^2^∙exp(−*a*(*r* − *b*)^2^, with *a* = 1.27 × 10^−3^ Å^−2^ and *b* = −25.3 Å, as shown in (**b**) for all employed ethylene glycol concentrations (0–92%). The normalization constant, *c*, is fixed by the condition that ∫*p*(*r*)*dr* = 1.

In our case, we were able to retrieve absolute values for the diffusion coefficient and distance distribution by a global analysis of all data points based on the Haas-Steinberg equation (HSE, Equation (18)), as described in the Material and Methods section. The result of the global optimization is shown in Figure 7a. The agreement of theoretical (black) and experimental (red and blue) data is remarkable, considering that only three parameters were freely adjustable in the optimization. The analysis yielded a diffusion coefficient of 55.4 Å^2^/ns in the absence of ethylene glycol, and a skewed Gaussian distance distribution, *p*(*r*) = *cr*^2^∙exp(−*a*(*r* − *b*)^2^, with *a* = 1.27 × 10^−3^ Å^−2^ and *b* = −25.3 Å (Figure 7b) at all solution conditions. Because the optimization could not directly provide confidence intervalls of the parameters of interest and because we have repeatedly expressed our skepticism, whenever very sharp values of diffusion coefficients were reported in the literature [13,36], we tested the robustness of the analysis and the results by also analyzing random subsets, limited to 15–35 points, of the entire set of 48 data points. This procedure and the analysis of 200 optimizations yielded *D* = 53.4 ± 6.0 Å, *a* = 1.28 ± 0.06 × 10^−3^ Å^−2^, and *b* = −25.7 ± 2.3 Å, which is very close to the results obtained from all 48 points.

End-to-end and site-to-site diffusion coefficients in linear polymers, mostly in peptides and proteins, have been published previously [15,34,36,37,38,39,40,41]. The reported values range from 4 Å^2^/ns [40] to 58 Å^2^/ns [34]. Most often, ensemble measurements on peptides and proteins [34,36,39,40] were analyzed on the basis of the Haas-Steinberg equation [14]. To dissect the contributions of the equilibrium distance distribution and diffusion requires a global analysis [42]. Haas et al. simultaneously analyzed the donor fluorescence kinetics as well as the decay kinetics of the acceptor optically excited through FRET [39,40,42]. Other proposals to carry out a global analysis included the use of an external quencher to modulate the donor lifetime [15], or the simultaneous analysis of the donor decay kinetics of peptides labeled with two different donors [34]. We challenged both attempts in previous works [13,36]. However, the corrected end-to-end diffusion coefficient, 58 Å^2^/ns, reported in reference [34] and measured for labeled (GS)_16_ peptides, is only slightly larger than our value of 55 Å^2^/ns for the (GS)_6_ peptides that we studied here. At short chain lengths, the diffusion coefficient is expected to grow with an increasing length of the probe-intermittent chain [36,39], but we can also expect that this effect will level off for chains of sufficient length; naturally, the parts of the chain closest to the probes exert the largest effect on probe diffusion.

In an outlook, we would like to emphasize that future applications of sdFRET are not limited to low-molecular weight polymers or short peptides. In investigations of protein folding processes, the ability to detect when two labeled chain sites approach each other closely can become crucial [43,44,45,46,47]. In regard to the structure and dynamics of synthetic polymers, the Duhamel group demonstrated the insight that can be gained from pyrene photophysics [48,49,50,51]. Along similar lines of synthesis, one could carry out a random copolymerization with added monomers carrying the optically active probes relevant in sdFRET. Polymers prepared with these probes usually allow the simultaneous application of a second photophysical method of CIFQ (collision-induced fluorescence quenching), as we demonstrated in two previous works [35,36].

## 5. Conclusions

Through a two orders of magnitude variation in parameter range, we have shown that the FRET diffusion enhancement depends symmetrically on the diffusion coefficient and the radiative donor lifetime. That this relationship holds has been expected on the grounds of theoretical considerations [13], however, it has never been consolidated in a systematic experimental analysis. The key result shown in Figure 5 also underscores and confirms that the FRET diffusion enhancement depends on the radiative and not on the measured donor fluorescence lifetime. In the case of a donor quantum yield of about 0.1, a quite common value, the radiative lifetime would exceed the measured lifetime by an order of magnitude ($\tau_{\mathrm{rad}}=\tau_{D}/\Phi_{D}$). If, in any FRET study, the measured donor lifetime is short, the FDE could still be high and would have to be acknowledged to arrive at meaningful information. Finally, as exemplified in this work, the *D*𝜏_rad_ symmetry can be employed to conclude on the impact of viscogenic coadditives on peptide chain dimensions. Measurements in the presence of a viscogen can even provide absolute parameters of chain dynamics and dimensions.
